# Balance Right in Multiple Sclerosis (BRiMS): a guided self-management programme to reduce falls and improve quality of life, balance and mobility in people with secondary progressive multiple sclerosis: a protocol for a feasibility randomised controlled trial

**DOI:** 10.1186/s40814-017-0168-1

**Published:** 2017-07-27

**Authors:** H. Gunn, J. Andrade, L. Paul, L. Miller, S. Creanor, C. Green, J. Marsden, P. Ewings, M. Berrow, J. Vickery, A. Barton, B. Marshall, J. Zajicek, J. A. Freeman

**Affiliations:** 1 Faculty of Health and Human Sciences, School of Health Professions, Plymouth University, Peninsula Allied Health Centre, Derriford Rd, Plymouth, PL6 8BH UK; 2 Faculty of Health and Human Sciences, School of Psychology, Portland Square Building, Drake Circus Campus, Plymouth, PL4 8AA UK; 3 School of Health & Life Sciences, Glasgow Caledonian University, Cowcaddens Road, Glasgow, G4 0BA UK; 4 Douglas Grant Rehabilitation Unit, Ayrshire Central Hospital, Kilwinning Road, Irvine, KA12 8SS UK; 5 Peninsula Schools of Medicine and Dentistry, Peninsula Clinical Trials Unit at Plymouth University (PenCTU), Room N16, Plymouth Science Park, Plymouth, PL6 8BX UK; 6 Medical Statistics, Peninsula Schools of Medicine and Dentistry, Room N15, Plymouth Science Park, Plymouth, PL6 8BX UK; 7 University of Exeter Medical School, Health Economics Group, University of Exeter, St Luke’s Campus, Exeter, EX1 2 LU UK; 8 NIHR Research Design Service (South West), Musgrove Park Hospital, Taunton, TA1 5DA UK; 9 NIHR Research Design Service, Peninsula Schools of Medicine and Dentistry, ITTC Building, Plymouth Science Park, Plymouth, PL6 8BX UK; 10 Cornwall, UK; 11 School of Medicine, Medical and Biological Sciences, University of St Andrews, North Haugh, St Andrews, KY16 9TF UK

**Keywords:** Secondary progressive multiple sclerosis, Exercise, Self-management, Mobility, Accidental falls, Balance, Quality of life, Feasibility randomised controlled trial

## Abstract

**Background:**

Impaired mobility is a cardinal feature of multiple sclerosis (MS) and is rated by people with MS as their highest priority. By the secondary progressive phase, balance, mobility and physical activity levels are significantly compromised; an estimated 70% of people with secondary progressive MS fall regularly. Our ongoing research has systematically developed ‘Balance Right in MS’ (BRiMS), an innovative, manualised 13-week guided self-management programme tailored to the needs of people with MS, designed to improve safe mobility and minimise falls. Our eventual aim is to assess the clinical and cost effectiveness of BRiMS in people with secondary progressive MS by undertaking an appropriately statistically powered, multi-centre, assessor-blinded definitive, randomised controlled trial. This feasibility study will assess the acceptability of the intervention and test the achievability of running such a definitive trial.

**Methods/design:**

This is a pragmatic multi-centre feasibility randomised controlled trial with blinded outcome assessment. Sixty ambulant people with secondary progressive MS who self-report two or more falls in the previous 6 months will be randomly allocated (1:1) to either the BRiMS programme plus usual care or to usual care alone. All participants will be assessed at baseline and followed up at 15 weeks and 27 weeks post-randomisation.

The outcomes of this feasibility trial include:

- Feasibility outcomes, including trial recruitment, retention and completion
- Assessment of the proposed outcome measures for the anticipated definitive trial (including measures of walking, quality of life, falls, balance and activity level)
- Measures of adherence to the BRiMS programme
- Data to inform the economic evaluation in a future trial
- Process evaluation (assessment of treatment fidelity and qualitative evaluation of participant and treating therapist experience)

**Discussion:**

The BRiMS intervention aims to address a key concern for MS service users and providers. However, there are several uncertainties which need to be addressed prior to progressing to a full-scale trial, including acceptability of the BRiMS intervention and practicality of the trial procedures. This feasibility trial will provide important insights to resolve these uncertainties and will enable a protocol to be finalised for use in the definitive trial.

**Trial registration:**

ISRCTN13587999.

## Background

Multiple sclerosis (MS) affects approximately 100,000 people in the UK [1], with an estimated cost of £1.4 billion/annum to the National Health Service (NHS) and wider society [2]. Although most people start with a relapsing-remitting (RR) disease course, approximately two thirds move to a secondary progressive phase within 8 years [3]. At this point, medical interventions are limited and progression is inevitable [4].

Surveys of people with MS (pwMS) consistently rank mobility as their highest priority and most important yet most challenging daily function [5]. Evaluation of treatments to improve mobility has also been highlighted as one of the top 10 MS research priorities by the James Lind Alliance [6]. Impaired balance and falls are common issues for people with secondary progressive MS (SPMS) and are an important contributory factor to mobility impairment [7, 8]. Approximately 70% of pwMS fall regularly [9, 10], at an average rate of >26 falls/person/year in SPMS [11]. More than 10% of these falls lead to injuries [12] and pwMS are three times more likely to sustain a fracture than the general population [13].

Falling and fear of falling have a profound impact on individuals, leading to activity curtailment, social isolation and a downward spiral of immobility, deconditioning and disability accumulation [14]. There are also substantial economic and social costs related to increasing immobility, impaired balance and falls in pwMS [15]. Costs of health and social care have been shown to increase steeply with increasing disease severity/immobility, underlining the importance of optimising safe mobility for as long as possible [16]. This is particularly relevant given evidence that pwMS are living longer, leading to a rising population living with the disease [17]. This has important implications for resource provision, as highlighted in a national audit of neurological services [18].

The importance of mobility and falls is further emphasised by their consistent prominence in policy documents for long-term neurological conditions [19]. Work suggests that falls may be an early marker of mobility deterioration associated with disease progression [9, 10]. Rehabilitation interventions which improve balance and physical activity and decrease the risk of falls may slow this deterioration, providing a persuasive argument to prioritise provision of effective physical management strategies. However, there is currently minimal evidence-based guidance to inform optimal mobility management and none to inform falls management in people with progressive MS. Whilst evidence is available for older people and those with other neurological conditions, research suggests that translating existing interventions to pwMS is likely to be ineffective [20, 21]. Small, limited duration studies have evaluated single elements of MS balance and falls interventions, individually demonstrating short-term improvements in mobility, balance or falls awareness [22–24], but these elements have not yet been implemented or evaluated collectively. Moreover, no studies have been confined to people with SPMS. This feasibility trial begins to address all these issues.

Healthcare policy prioritises the need to empower and support patients to self-manage [25]. ‘Balance Right in MS’ (BRiMS) is an innovative evidence-based, user-focused, self-management exercise and education programme, designed to improve safe mobility and reduce falls for pwSPMS. It is critical to assess the delivery of this programme and proposed evaluation methods prior to undertaking a definitive trial to assess its effectiveness and cost effectiveness.

Following Medical Research Council (MRC) Guidelines [26], this feasibility trial will aid the planning of an anticipated definitive, multi-centre randomised controlled trial (RCT) which will compare BRiMS plus usual care with usual care alone in improving mobility and quality of life (QoL), and reducing falls in people with SPMS. This feasibility trial will provide the necessary data and operational experience to inform the conduct and finalise the design of a definitive trial so that it can be successfully delivered with confidence. Ultimately, this will add significantly to the evidence by reporting results of a robust RCT of a manualised, complex intervention.

## Methods

### Trial design

**Fig. 1:**
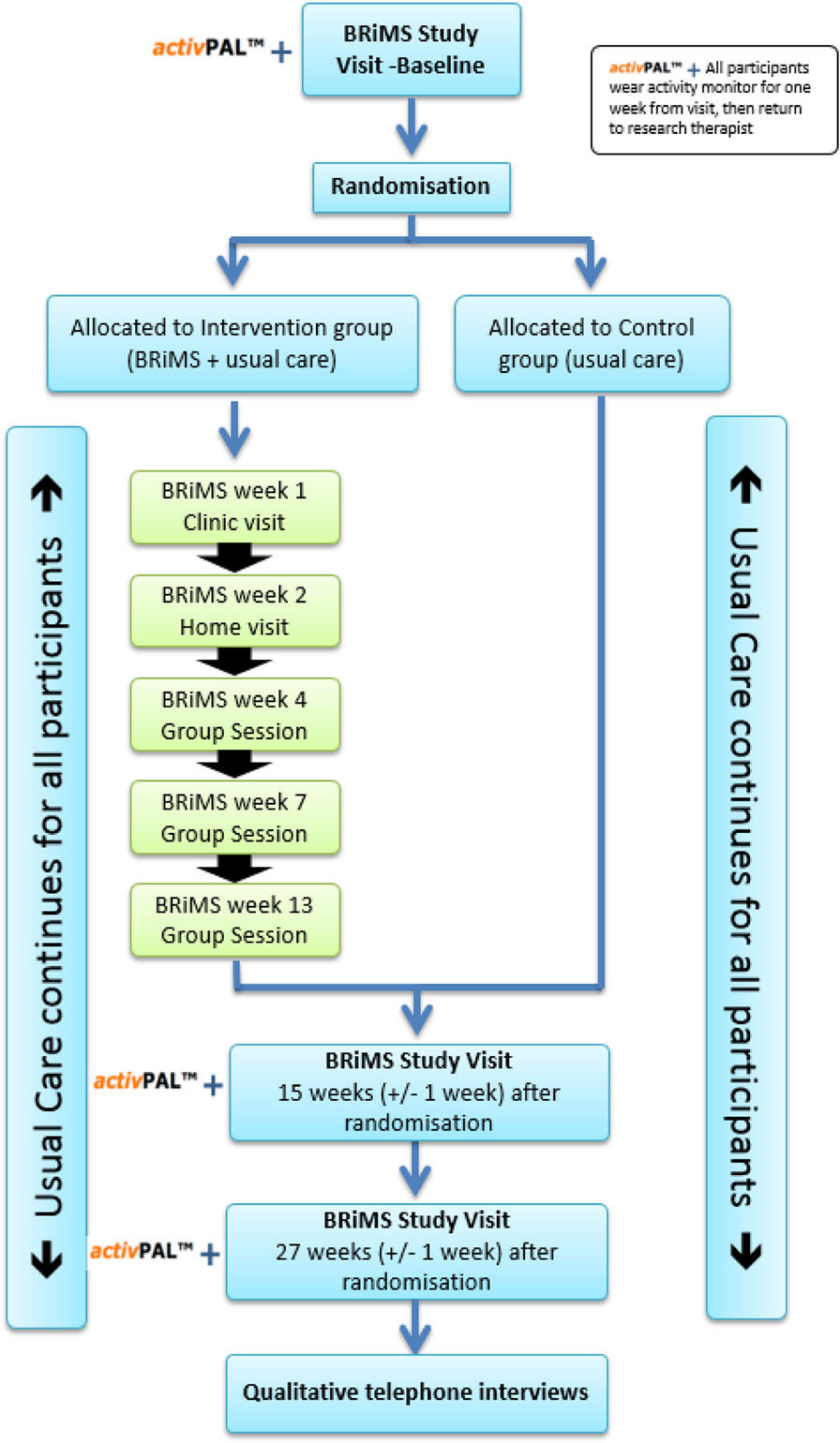
Participant pathway

This is a pragmatic, multi-centre, feasibility RCT with blinded outcome assessment. Figure 1 shows the planned participant pathway.

### Trial settings

Four healthcare sites will be involved in this multi-centre RCT, which is based in two geographical regions of the UK: South West Peninsula (England) and Ayrshire (Scotland). A full list of study sites is available via www.brims.org.uk.

### Participants

#### Sample size

As this is a feasibility trial, the more usual sample size calculation, based on considerations of power for detecting a between-group clinically meaningful difference in a primary clinical outcome, is not appropriate [27]. Instead, the aim is to provide robust estimates of the likely rates of recruitment and follow-up, as well as provide estimates of the variability of the proposed primary and secondary outcomes to inform sample size calculations for the planned definitive trial. Therefore, we aim to recruit a total of 60 participants across the two regions (40 in the South West and 20 in Ayrshire) over 6 months. An estimated 240 people will need to be screened to achieve this sample size. From other studies in similar settings, we anticipate that retention rates will be approximately 80% [28, 29]. With our intended sample size of 60 participants, we will be able to estimate the overall retention rate with precision of at least ±13%, and if the 6-month follow-up rate is around 80%, this estimate will have precision of around ±10%. Assuming a non-differential 6-month follow-up rate of 80%, this should provide follow-up outcome data on a minimum of 24 participants in each of the allocated trial arms.

### Inclusion criteria

The trial population will comprise individuals with a confirmed diagnosis of MS as has been determined by a neurologist according to revised McDonald’s criteria [30], and who are in the secondary progressive phase.

Participants will:

- Be aged ≥18 years
- Be willing and able to understand/comply with all trial activities
- Score ≥4.0 ≤ 7.0 on the Expanded Disability Status Scale, i.e. people who have some mobility impairment, but who are ambulant for at least a proportion of the time
- Self-report two or more falls in the past 6 months
- Be willing and able to travel to local sites for blinded outcome assessments and BRiMS programme sessions
- Have access to a computer or tablet and to the internet

### Exclusion criteria

Potential participants will be excluded if they:

- Have relapsed/received steroid treatment within the last month (patient-reported relapse is defined as ‘the appearance of new symptoms, or the return of old symptoms, for a period of 24 h or more—in the absence of a change in core body temperature or infection’) [31]
- Have had any recent changes in disease-modifying therapies; specifically, if they have ever had previous treatment with alemtuzemab; are within 6 months of ceasing nataluzimab; or are within 3 months of ceasing any other MS disease-modifying drug
- Have participated in a falls management programme within the past 6 months
- Report co-morbidities which may influence their ability to participate safely in the programme or are likely to impact on the trial (e.g. uncontrolled epilepsy).
- Are participating in a concurrent interventional study

Identification and recruitment of participants will be via several routes, including identification by healthcare professionals, screening MS databases and promotion via MS support groups and newsletters. This will be supported by National Institute for Health Research (NIHR) Clinical Research Network staff at each site.

### Randomisation and allocation concealment

The inclusion of group-based elements as part of the intervention necessitates the confirmed participation of a sufficient number of participants within a recruiting site before randomisation occurs. There are four sites where the intervention will be delivered. Once recruited, participants will ideally be randomised in blocks of 10, but the process can accommodate some flexibility within the limits 8–12 participants in each block. Randomisation will be undertaken when a sufficient number of individuals from a recruiting site have consented, indicated that they are able to attend the same BRiMS group (location, timing, should they be randomised to receive it), and complete baseline data have been collected. The decision to declare a block complete will be made by the research therapist in collaboration with the Trial Co-ordinator, Chief Investigator and local UK Clinical Research Collaboration registered Clinical Trials Unit (CTU) (Registration number 31). Randomisation will be undertaken a minimum of 3 and a maximum of 7 days prior to the commencement of the BRiMS programme delivery (for each block).

When the block size from a recruiting site consists of 8–12 participants, the participants will be randomised to the intervention or control group, using block simultaneous randomisation. The randomisation will be 1:1 when the block consists of an even number of participants, and when the block consists of an odd number of participants, the allocation ratio will be in favour of the intervention group in order to maximise recruitment potential and learning opportunities in this feasibility trial. Participants in a block will be numbered in the order in which they were first entered onto the trial website. The randomisation process will follow a strict and auditable protocol. Randomisation will take place after completion of all baseline assessments by the CTU Trial Manager via a secure web-based system. The randomised allocations will be computer-generated by the CTU in conjunction with an independent statistician, in accordance with the CTU’s standard operating procedure. The randomisation list and the programme that generated it will be stored in a secure network location within the CTU, accessible only to those responsible for provision of the randomisation system.

After randomisation has taken place, an automatic email will be sent by the CTU to the NHS Therapist leading the BRiMS programme locally and to the relevant Principal Investigator to notify them of each participant’s allocated group. Notification that randomisation has taken place (but *no* details regarding individual participant’s allocated group) will also be sent to the relevant research therapist and to the CI.

Access to the randomisation code and allocation list will be confined to the CTU data programmer; no one else in the trial team will be aware of allocated trial arms until formal randomisation is completed, hence maintaining effective concealment. Following randomisation, only appropriate members of the trial team will be aware of participants’ allocations to intervention or control group; the blinded research therapists will *not* have access to treatment allocation.

### Blinding

Due to the nature of the intervention, trial participants and treating physiotherapists are unable to be blinded. However, the assessors who are undertaking the outcome assessments will be blinded to participant allocations. The initial baseline assessment will be undertaken, following written informed consent obtained by the research therapist, prior to randomisation. Every effort will be made to ensure the two follow-up assessments (at 15 and 27 weeks post-randomisation) remain blinded. At each assessment time point, the assessor will be asked to record if they were un-blinded to group allocation, and if so, the reasons for this.

### Interventions

The BRiMS programme is delivered as a 13-week therapy-led personalised education and exercise intervention. It is structured to maximise the development of self-efficacy and support participant engagement. BRiMS aims to address modifiable fall risk factors such as poor balance and mobility and enable self-management by the use of individualised mobility, safety and falls risk management strategies.

The development of BRiMS has been informed by the MRC framework for the development and evaluation of complex interventions [26] through a comprehensive programme of research [9, 11, 32–34] and input from internationally recognised experts [23, 35].

BRiMS includes a strong focus on home-based activities, supported by online resources and three group sessions interspersed over the duration of the programme. The programme also includes two ‘one-to-one’ sessions (in weeks 1 and 2) to enable individualised assessment, goal planning and development of exercise plans. A home-based online work package overarches the programme, supporting both educational and exercise components and enabling participants to personalise the programme and apply the activities in their daily lives from the outset (www.brims.org.uk). Developing and supporting motivation is addressed throughout by using innovative functional imagery techniques [36] to supplement established motivational techniques.

**Fig. 2:**
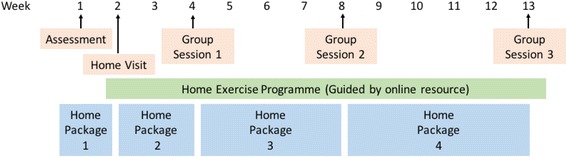
BRiMS programme activities

Whilst the BRiMS programme is manualised, it is structured to enable tailoring of the components to meet the individual needs of participants (Fig. 2).

The BRiMS education component aims to improve exercise self-efficacy and enhance the individual’s knowledge and skills about falls prevention and management [37]. This is delivered through a mix of home and group activities embedded throughout the course of the programme. It utilises group brainstorming, problem solving and action planning [38] and applies the principles of cognitive behavioural therapy (CBT). During group sessions, peer modelling, vicarious learning, social persuasion and guided mastery are used to boost self-efficacy [39]. Activities which encourage the setting and imagery of short-term exercise goals are employed to boost desire to achieve them, and images of failure are ‘rescripted’ [40].

The BRiMS exercise component supports the participant to undertake at least 120 min of gait, balance and functional training per week. It has been designed to be predominantly home-based, with exercise planning and progression undertaken in partnership between the participant and programme leader. The group sessions include exercise activities to encourage peer support and problem solving. Additionally, BRiMS integrates an online exercise prescription resource [41] to support and guide home-based practice (www.webbasedphysio.com).

### Usual care

This feasibility trial will use a usual clinical care control group. Whilst usual care varies across the country [42], in those with SPMS, physiotherapy input is generally provided when an event has caused a significant deterioration in the person’s ability to function (e.g. a respiratory infection or an injurious fall). The standard physiotherapy care pathway usually comprises short intermittent episodes of face-to-face intervention. Typically, presenting problems are managed (e.g. providing mobility aids, a written home exercise programme and advice) rather than focusing on the promotion of long-term self-management strategies. In this trial, the usual care received (including health/social care interventions and medications) will be recorded within the health economic assessment of resource utilisation. In this feasibility study, the therapists providing the intervention are not involved in providing the control group with ‘usual care’, thereby avoiding potential contamination.

### Data collection and outcome measurements

Participant data will be collected during face-to face visits and through the return of postal diaries (see Table 1 for details). All participants will attend for three trial assessment visits undertaken by the blinded research therapists at baseline, 15 weeks (±1 week) and 27 weeks (±1 week) after randomisation. These dates allow the delivery of the pre-scheduled BRiMS programme to be completed prior to the first follow-up assessment. Deviations from this schedule will be monitored and reported on a protocol deviation form.

**Table 1:** Data collection and outcome measures

Outcome measure	Baseline		15 weeks post-randomisation (±1 week)	27 weeks post-randomisation (±1 week)
Demographics and history	x	Randomisation to intervention or control		
Provide falls diaries	x		Returned by post every 2 weeks	
EDSS	x			
MSWS-12vs2.0	x		x	x
MSIS-29vs2.0	x		x	x
EQ-5D-5L	x		x	x
Falls frequency	x		x	x
Activity monitoring	1 week		1 week	1 week
2-minute walk test	x		x	x
Mini-BEST	x		x	x
Functional Reach Test	x		x	x
Falls Efficacy Scale	x		x	x
Community Participation Indicators	x		x	x
Participant Resource Use questionnaire	x		x	x
Adverse events			x	x
Qualitative interviews				x

The following data will be collected during the trial:

A. Feasibility outcomes
  Data from screening, recruitment and follow-up logs will be used to generate realistic estimates of eligibility, recruitment, consent and follow-up rates.
B. Clinical outcome measures
  Standardised clinician-rated and patient self-reported clinical outcomes, which have demonstrated good reliability and validity in people with MS, will be measured. Further, in the main, these measures have been widely used in MS interventional studies, which will enable comparison between studies.
  1. Possible primary outcomes for the definitive trial
*Walking*—Multiple Sclerosis Walking Scale–12 item (MSWS-12) Version 2.0 [43].
*Health-related quality of life EuroQoL* (EQ-5D-5L) [44] and the 29-item *Multiple Sclerosis Impact Scale* (MSIS-29) Version 2.0 [45], which have been specified for use in health economic analyses in MS studies [46].
  2. Possible secondary outcomes for the definitive trial
*Falls frequency and injury rates*
    Falls will be defined as “an unexpected event in which you come to rest on the floor or ground or lower level” [47]. In line with best practice guidance, the number of falls, injurious falls and associated use of medical services will be recorded prospectively using a patient-completed daily diary returned to the CTU in a FREEPOST envelope on a fortnightly basis [48].
*Activity level* using an activity monitor (activPAL™, Paltechnologies Ltd, Glasgow) [49].
*Walking capacity* using the two-minute walk test (fastest speed) (2MWT). This has been recommended as the standard objective walking test to be used in MS interventional studies [50].
*Balance* using the Mini-Balance Evaluation Systems Test (Mini-BEST) [51] and the Functional Reach Test (forwards and lateral) [52–54].
*Fear of falling* using the 16-item self-report Falls Efficacy Scale (International) (FESi) [55]. This has been recommended as the standard objective measure of fear of falling by the Prevention of Falls Network Europe [47].
*Community integration* using the self-report Community Participation Indicators (CPI) [56]. This has been recommended as an objective measure of participation for use in falls prevention studies by the International MS Falls Prevention Research Network [57].
C. Measures of adherence
  Attendance at the five face-to-face sessions will be recorded; adherence will be calculated as a percentage. Engagement in the home-based programme (BRiMS online exercise package and educational packages) will be recorded based on the participants' web-based activity and participant reported information.
  This information, alongside the data obtained from qualitative interviews with participants (see E below) will be used to evaluate levels of adherence and to determine whether any amendments to the programme are required to improve engagement.
D. Economic evaluation
  Methods for the collection of resource use, cost, and outcome data will be developed and tested in preparation for an economic evaluation alongside a full trial. Data on resource use associated with the setup and delivery of the BRiMS intervention will be collected via within trial reporting, including participant level contact and non-contact time for staffing input on delivery, equipment and consumable costs, training and supervision. Data on health and social care resource use will be collected at participant level using a Participant Resource Use (RU) questionnaire, developed for this trial [58]. The EQ-5D-5L will be used to estimate quality-adjusted life-years (QALYs), and is the expected primary economic endpoint (cost per QALY) in any future evaluation. The MSIS-8D [59, 60], an MS-specific preference-based (QALY) measure, will also be used, as this is expected to be of value in future sensitivity analyses.
E. Process evaluation
  Process evaluation is a key part of the intervention development process and will be guided by the MRC Process Evaluation of Complex Interventions Guidelines [61] and the National Institute of Health Behaviour Change Consortium framework [62].

1. Standardisation and fidelity of the intervention
  Two research therapists (employed specifically for the trial) will undertake the blinded assessments using standardised written protocols. Treating therapists from each site will perform the interventions as part of their NHS physiotherapy role. For this feasibility trial, all treating therapists will undertake a training session as a group. They will receive a therapist manual that provides a clear structure to each session, details the session content and provides sample scripts. All treating physiotherapists have access to the programme website (containing comprehensive reference materials and a closed therapist discussion forum) to optimise fidelity to the intervention content and approach. Treatment fidelity of a random sample of a minimum of 25% of the delivered sessions will be assessed using audio recordings of the session. This sample will include at least two recordings of each session type (1:1 assessment, home visit and group sessions) and at least one session from each treating therapist. The assessment of fidelity will be undertaken by two members of the research team who are independent from the intervention delivery, using a checklist which has been informed by the Dreyfus System for Assessing Skill Acquisition [63] and an adaptation of the Motivational Interviewing Treatment Integrity scale [64]. Both reviewers will initially meet to discuss the fidelity assessment process and their expectations for each element of the checklist. They will then independently rate the same recording and compare and moderate their assessments prior to undertaking further reviews. Any uncertainties in further reviews will be resolved through discussion.
*Safety monitoring*
  Throughout the trial, all possible precautions will be taken to ensure participant safety and wellbeing. Participants will be monitored for adverse events and serious adverse events (defined according to the Medicines for Human Use (Clinical Trials) Regulations, 2004) [65] via completion of their daily diaries and during follow-up assessments. Participants will be asked to report all adverse events in their diaries, whether they are thought to be related to the intervention or not. Diaries will be reviewed on receipt for reports of adverse events and responded to according to pre-defined adverse event and serious adverse event reporting procedures.
*Retention rates and withdrawals*
  Each participant has the right to voluntarily withdraw from the trial at any time, without repercussions. This is distinct from participants in the intervention group terminating their involvement in the BRiMS programme.
  - Discontinuation of the intervention
    Participants in the intervention group may choose to discontinue the BRiMS programme, or may do so on the recommendation of a health professional, for example following an adverse event. Where appropriate, such participants will be asked to continue to attend blinded assessments as per protocol if this is feasible.
  - Withdrawal from the trial
    Any participant may at any time after they have consented decide that they no longer wish to be part of the trial. This may be through personal choice (i.e. they withdraw their consent) or in consultation with a health professional, for example where it becomes impossible to provide outcome data or comply with any other trial procedures for whatever reason. In addition, a participant may be withdrawn following a significant protocol deviation, such as being randomised in error. In this event, the decision as to whether they should be removed from the trial completely or retained on an intention to treat (ITT) basis will be made through an independent adjudication by the Trial Steering Committee (TSC) who are blinded to group allocation [68].
2. Qualitative evaluation
  The qualitative evaluation aims to:
  Assess the acceptability of the trial methods (both trial arms)
  Evaluate the acceptability of the intervention and identify possible adaptations
  Identify the components of the intervention perceived to be effective.

One-to-one telephone interviews with trial participants and a telephone focus group [66] with treating therapists will be undertaken by the regional BRiMS trial coordinators at the completion of the programme. A purposive sample of 10 participants will include people from different regions, different BRiMS intervention groups and a sample of control arm participants. Participants will be contacted and a mutually convenient time agreed to undertake a telephone interview within 2 weeks of the completion of their final trial visit. All treating therapists will be invited to participate in the telephone focus group which will be convened within 1 month of the completion of the final BRiMS programme delivery. All interviews will be digitally recorded and transcribed verbatim. The researchers will employ a reflexive approach throughout, utilising research diaries, field notes and critical reflection [67].

### Data analysis

In keeping with the aims of a feasibility study, a detailed statistical analysis plan will be developed and approved by the Trial Steering Committee, prior to final database lock and analyses. For the final analysis, the trial statistician will be presented with a database by the CTU containing a group code for each participant but not identifying which group is which; only after the primary analyses will the two groups be identified.

The analyses of the quantitative data will be in two stages, with data summarised according to participants’ allocated trial arm. All analyses will be undertaken and reported according to the recently published CONSORT guidelines for pilot and feasibility trials [68].

*Stage 1* will summarise the feasibility outcomes: data from screening, recruitment and follow-up logs will be used to generate realistic estimates of eligibility, recruitment, consent and follow-up rates and presented in a CONSORT flowchart. In addition, adherence data (e.g. session attendance and exercise adherence) will be used to contribute to the evaluation of the acceptability and concordance to the BRiMS programme. Completion rates will be estimated for each of the outcome measures at each time point. All estimates will be accompanied by appropriate confidence intervals, to allow assumptions to be made in the planning of the definitive trial. The baseline characteristics of individuals lost to follow-up will be compared to those who complete the feasibility trial to identify any potential bias.

*Stage 2* will summarise the clinical outcomes data at each time point. As it is inappropriate to use feasibility trial data to formally test for between-group treatment effects, the analyses will primarily be of a descriptive nature [27, 69]. The CONSORT extension for reporting of pilot and feasibility studies [68] and the CONSORT extension for reporting of patient-reported outcomes [70] will be followed. Descriptive statistics of the clinical outcomes data will be produced for each trial arm. Interval estimates of the potential intervention effects, relative to usual care only, will be produced in the form of a 95% confidence interval, to ensure that the effect size subsequently chosen for powering the definitive trial is plausible, but no formal hypothesis testing will be undertaken [27].

### Qualitative analysis

The qualitative analysis will employ a constructivist paradigm, described as an approach which allows the co-creation of understandings by respondent and researcher [71]. The qualitative data will include transcripts from one-to-one participant interviews, and the health professional telephone focus group.

Anonymised transcribed data will be entered into NVIVO software (QSR International, Southport, UK). A pragmatic process of data immersion, coding and generation of initial themes will then be undertaken [72]. Subsequently, these themes will be refined in discussion with research team members to maximise credibility of the process [73]. The rigour of the qualitative analysis will be maximised through use of a range of techniques, including exploration of contradictory evidence, respondent validation, and constant comparison [74]. In addition, interview and focus group participants will be invited to review an initial draft to ensure the analysis represents an accurate overview of participants’ views, experiences and recommendations. Once this has been verified, the data will be used to (where necessary) revise the BRiMS Operational Manual and Trial Procedures.

### Determining progression to the full trial

We shall progress to a full trial application if minimum success criteria are achieved in key feasibility aims and objectives, or if we can identify solutions to overcome any identified issue. These criteria will be finalised in discussion with the Trial Steering Committee, but are likely to include:

- A minimum of 80% recruitment of the intended 60 participants within the 6-month recruitment window
- A minimum of 80% completion rate of key outcome measures (including follow-up)

### Data management, audit and monitoring

The CTU will be responsible for data management for the study. Data will be recorded on study-specific data collection forms by the blinded assessors, and on self-completion forms by study participants. Completed forms will be passed to the CTU and entered onto a secure web-based database. All data will be double entered and compared for discrepancies. Discrepant data will be verified using the original paper data sheets.

Data will be collected and stored in accordance with the Data Protection Act 1998 and will be accessible for the purposes of monitoring, auditing, or at the request of the regulatory agency.

### Trial oversight

There are three committees involved in the setup, management and oversight of this trial: the Trial Management Group (TMG), the Trial Steering Committee (TSC) and the Data Monitoring Committee (DMC).

The TMG comprises those individuals involved in the development of the protocol and the day-to-day running of the study. The responsibility of this group is to ensure all practical details of the trial are progressing, and everyone within the trial understands them. This includes monitoring adverse events, recruitment and attrition rates, the project timeline and finances. It will also include responsibility for the release of the trial results and publications. The TMG will meet approximately monthly.

The TSC is responsible for overseeing the conduct of the trial and comprises a group of experienced trialists with majority independent representation. The TSC will meet before the start of the trial and subsequently at least annually. In addition, the TSC and DMC will receive a quarterly report of adverse events, and a telephone conference/additional face-to-face meeting will be instigated by the chair of either group, or the chief investigator (CI) should any issues need to be discussed.

The DMC comprises an independent statistician and two experienced clinical trialists, one of whom will be the chair. This committee will be independent of the study organisers and the TSC; the DMC will maintain the interests of trial participants, with particular reference to safety, and will report to the chair of the TSC. It is anticipated that the members will meet once to agree terms of reference and subsequently at a schedule to be agreed with the TSC.

### Ethics

The trial will be conducted in accordance with the Declaration of Helsinki, 1996 [75]; the principles of Good Clinical Practice, and the Department of Health Research Governance Framework for Health and Social Care, 2005 [76]. All ethical approvals will be in place prior to the commencement of trial recruitment activities (see declarations section).

### Dissemination plan

The results of this feasibility trial will inform the design of the anticipated definitive trial, rather than directly inform clinical decision making, since clinical and cost effectiveness cannot be determined at this level. Hence, dissemination, regardless of outcome of this feasibility trial, will focus on publication of the feasibility outcomes, and related methodological issues, in open access peer-reviewed journals.

On completion, the full study report will be accessible on the study website (www.brims.org.uk) and via the funding body website, as will the full protocol. This protocol (Version 3.0, dated 07 Dec 2016) has been written in line with SPIRIT Guidelines [77]. Similarly, the Consolidated Standards of Reporting Trials (CONSORT) [68, 78] and the Template for Intervention Description and Replication (TiDIER) Guidelines [79] will be reviewed prior to submitting future publications of the trial results. Authorship of articles will be by the study team; professional writers will not be used.

Results of this feasibility trial will be presented at national and international conferences to engender enthusiasm for the potential future trial. Summaries will be posted on to the websites/newsletters of the organisations who were involved in the recruitment process. In addition, all participants will be offered a lay summary of results and a clinically oriented summary will be provided to recruiting centres. A key output will be an application for funding for a definitive trial, if the results of the feasibility trial meet the criteria for progression.

## Discussion

The importance of developing interventions to support pwMS to maintain their mobility and manage falls has been highlighted by service users and providers, and in practice guidelines [1, 6, 19]. The BRiMS intervention has been developed with the aim of addressing this important issue; however, a full evaluation of its effectiveness is essential to inform evidence-based clinical decision making. Best practice guidance emphasises the need to thoroughly test the feasibility and acceptability of both interventions and trial evaluation procedures prior to undertaking a full-scale assessment of effectiveness [26]. This feasibility trial will provide important insights into the practicality of running a full-scale trial to evaluate BRiMS, including providing estimates of: recruitment, attrition, adherence, baseline scores, standard deviations and completion rates of the measures. It will also enable us to assess the acceptability of the intervention and of participating in the trial from the participant and health professional perspective, and the process of delivering BRiMS, to finalise a protocol for use in the definitive trial. Whilst the trial has been developed according to best practice guidance, the methodology is not without potential limitations. For example, the use of active and attention-matched control groups has been debated in the literature [80, 81]; in this trial, the lack of evidence to inform the selection of an active comparator, along with the cost implications of including a third attention-matched group, led to the pragmatic decision to utilise a usual care control group.

One potential scheduling issue that we aim to test is the feasibility of the relatively short (minimum 3 days) timescale between randomisation and commencement of the BRiMS programme for those allocated to the intervention group, necessitated by the group elements within the BRiMS programme. Whilst considerable thought has been given to minimising this challenge, for example by pre-scheduling the BRiMS programme dates, qualitative feedback from participants and treating therapists will be important to finalise this aspect of a future full-scale trial.

This trial specifically targets people with SPMS, whose MS type and level of impaired mobility often makes them ineligible to participate in clinical trials, and for whom medical intervention is limited. Whilst we have estimates of potential recruitment and retention rates from other studies of similar interventions, these trials included participants with a range of MS sub-types. Therefore, it will be important to assess whether these estimates are appropriate for people with SPMS who may have more health issues which could impact on their participation in trials.

### Trial status

Recruitment commenced mid-January 2017.

## Abbreviations

2MWT: Two-minute walk test
CONSORT: Consolidated Standards of Reporting Trials
CPI: Community Participation Indicators
CTU: Regional Clinical Trials Unit
DMC: Data Monitoring Committee
FESi: Falls Efficacy Scale (International)
FRT: Functional Reach Test forwards/lateral
HRA: Health Research Authority
ISRCTN: International Standard Randomised Controlled Trials Number
Mini-BEST: Mini-Balance Evaluation Systems Test
MRC: Medical Research Council
MS: Multiple sclerosis
MSIS-29vs2.00: MS Impact Scale (29 item) version 2
MSWS-12vs2.00: MS Walking Scale (12 item) version 2
NHS: National Health Service
NIHR: National Institute for Health research
NRES: National Research Ethics Scheme
pwMS: People with MS
QALY: Quality-adjusted life-year
QoL: Quality of life
RCT: Randomised control trial
REC: Research Ethics Committee
SPMS: Secondary progressive MS
TiDIER: Template for Intervention Description and Replication
TMG: Trial Management Group
TSC: Trial Steering Committee
